# Automatic object detection for behavioural research using YOLOv8

**DOI:** 10.3758/s13428-024-02420-5

**Published:** 2024-05-15

**Authors:** Frouke Hermens

**Affiliations:** Open University of the Netherlands, Heerlen, The Netherlands

**Keywords:** Surgical tool tracking, Automatic object detection, YOLO, Behavioural analysis

## Abstract

Observational studies of human behaviour often require the annotation of objects in video recordings. Automatic object detection has been facilitated strongly by the development of YOLO (‘you only look once’) and particularly by YOLOv8 from Ultralytics, which is easy to use. The present study examines the conditions required for accurate object detection with YOLOv8. The results show almost perfect object detection even when the model was trained on a small dataset (100 to 350 images). The detector, however, does not extrapolate well to the same object in other backgrounds. By training the detector on images from a variety of backgrounds, excellent object detection can be restored. YOLOv8 could be a game changer for behavioural research that requires object annotation in video recordings.

## Introduction

In behavioural research, a common part of the analysis process is the annotation of videos. For example, to determine where people look when watching videos or engage in day-to-day activities, a regions of interest analysis can be performed on eye-tracking data to determine how long participants look at particular objects or people in a scene (e.g. Hermens, 2017; Gregory et al., 2015; Kuhn et al., 2016; Land et al. ; 1999). This requires detecting when the point of gaze of the participant enters a bounding box or a polygon region around a target object or person, which will need to be defined for each video frame. Likewise, studies that examine how participants handle day-to-day objects require the annotation of particular objects in videos of participants, for example, to determine where on a plunger (Cohen & Rosenbaum, 2004), a bowl (Hermens et al., 2014), a glass or a bar (Knudsen et al., 2012) participants place their hand when grasping these objects. A third situation involves surgical tool tracking in the context of studies of eye–hand coordination in surgeons performing key-hole surgery (for reviews, see Hermens et al., 2013; Tien et al., 2014; Gil et al., 2022). To obtain a measure of the skill of a surgeon, the position of the instrument in the video images can be analysed on their own or in relation to the direction of gaze (Ahmidi et al., 2010, 2012).

The task of drawing a bounding box around an object of interest is known as object detection within the computer vision literature. It is one of several methods that computer algorithms can now perform with high accuracy. Other tasks include image classification (Himabindu & Kumar, 2021, e.g. deciding whether an image contains a White or a Black person), image segmentation (Minaee et al., 2021, e.g. drawing a contour around a person), pose estimation (Chen et al., 2020, e.g. localising the position of feet, knees, hips, and shoulders of a person in an image), and object tracking (Chen et al., 2022, i.e. object detection while also tracking the identity of a person or object in the image). The accuracy of these techniques has improved substantially over the past years (Feng et al., 2019) due to improved algorithms, improved technology (particularly the introduction of graphical processing units, GPUs), and larger annotated datasets (e.g. Deng et al., 2009; Yang et al., 2015).

Until recently some level of programming skills was required to apply these computer vision methods, which can be an issue for behavioural researchers. This changed recently with the Ultralytics (Jocher et al., 2023) package that is easy to install and use for all of the above computer vision tasks. Applying computer vision methods with the Ultralytics packages means installing the package, sorting files into the appropriate folder structures, providing commands from the command line (Jocher et al., 2023) and saving the results.

Some object detection tasks can be performed with pre-trained models. This object detection can be performed on a standard personal computer (e.g. with an i5 processor and 8Gb of RAM). Pre-trained models tend to be based on the COCO dataset (Lin et al., 2014) that contains 80 types of objects. When the aim is to detect an object that is not in these pre-trained models (e.g. a plunger, a surgical instrument), a new model needs to be trained. For such training, a computer with a graphical processing unit is recommended (Google currently offers free processing time on their Colab servers, Bisong and Bisong, 2019).

To train a new object detector, a set of examples is needed. This involves finding images of the object and drawing bounding boxes around the object in each of these images, for example using the labelMe software (Wada, 2018). Training a new object detector often starts with a pre-trained network (often based on the COCO dataset, Lin et al., 2014) that takes advantage of pre-trained weights for the feature recognition stages of object recognition, a process known as transfer learning.

Typical object detection often involves highly variable contexts (e.g. various outdoor scenes, different weather and lighting conditions) and highly variable objects (e.g. various shapes, sizes and colours of cars and trucks). A common strategy is to use an already-existing dataset of annotated images (e.g. Yang et al., 2015; Krizhevsky et al., 2009; Deng et al., 2009). Using such an existing dataset may not always be a feasible strategy for objects used in the lab, as existing datasets may not contain the class of object that you may wish to detect (e.g. a plunger, Cohen and Rosenbaum, 2004). The question therefore arises what is required to train a new object detector for the object(s) under study.

Behavioural research may have an important advantage in this context. Experiments are often done in a much more controlled setting than found in typical object detection. Participants are all tested in the same lab, under the same lighting conditions, with the same camera viewpoint, manipulating the same object. Guidelines on, for example, how many images to annotate for training from typical object detection contexts may therefore not automatically apply to object detection in behavioural research (particularly when conducted in the lab).

**Fig. 1:**
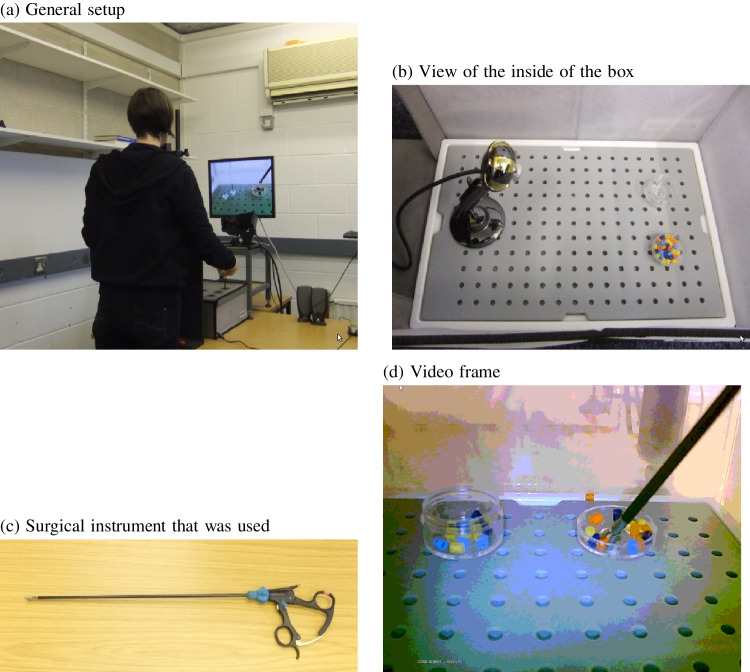
Participants stood in front of a surgical simulator box with their chin in a chin rest (**a**). Inside the surgical box were two dishes containing beads and a webcam (**b**). Participants entered a surgical instrument (**c**) into one of the holes in the top of the box and moved as many beads from one dish to the other in the three allotted minutes while the webcam recorded the view inside the box (**d**)

A recent study examined the effects of the number of annotated images used to train an object-detector for playing cards. For object detection, the authors used the You Only Look Once (YOLO) algorithm and two of the older versions of this algorithm (YOLOv1 and YOLOv2, i.e. versions 1 and 2). They found that precision and recall (measures of the accuracy of detection) improved until 300 images and with at least 300 training epochs (Li et al., 2018). This suggests that a relatively small number of annotated images may suffice for reliable object detection, but it is unclear how these results extend to more recent object detectors (e.g. Jocher et al., 2023, 2023), which reports substantially improved object detection over the earlier versions (for an overview, see Jiang et al., 2022).

The present study will therefore examine the required conditions to train object detectors for behavioural research. It will focus on YOLOv8 because this version is particularly easy to use compared to other object detectors. The main set of experiments in the present study will focus on surgical tool detection, as this type of object detection has substantial interest in the medical community and studies have demonstrated that accurate detection can be achieve with earlier YOLO versions (e.g. Choi et al., 2017; Choi et al., 2021; Wang et al., 2021). The present study will make use of images of a surgical tool inside a simulator box to mimic the low variability contexts typically found in lab-based studies (the experiment from which these images were sampled will be described in the Methods section below).

To examine how well the results generalise to other objects, the YOLOv8 detector will also be applied to a second dataset in which participants moved a transparent bowl between rings (Hermens et al., 2014). This particular application could pose an additional challenge to the algorithm due to the transparency of the bowl, additional occlusion, and the low image quality of this older dataset.

In the experiments, the following research questions will be addressed: (1) How many annotated images are needed to train an object detector in a low variability setting? (2) How well does the object detector perform on unseen videos of the object? (3) Does the YOLO version and the pre-trained model size affect performance of the detector? (4) Does an object detector trained for one background perform adequately when used for the same object but a different background? (5) If performance drops with a change of background, does it suffice to train a new detector in a new context? (6) How well does an object detector perform when trained on different contexts and how many images are needed per context? (7) How do results depend on the random selection of images for training? (8) Are similar image set sizes needed for other types of objects?

The present study will be unable to address all possible questions regarding the training of object detectors as it only focuses on YOLOv8 from the Ultralytics package and a limited set of research questions. It will also focus on object detection and not on other computer vision tasks, such as image segmentation and pose estimation. An important aim of the present article will therefore also be to illustrate how to conduct ‘experiments’ with computer vision software, very much like a behavioural study. The computer vision software is treated as a black box receiving stimuli and producing responses and the aim is to uncover the relationship between these inputs and responses and build a mental model of the black box. This general approach can be used for other types of computer vision tasks, other algorithms for the same task, or other objects of interest for detection.

**Fig. 2:**
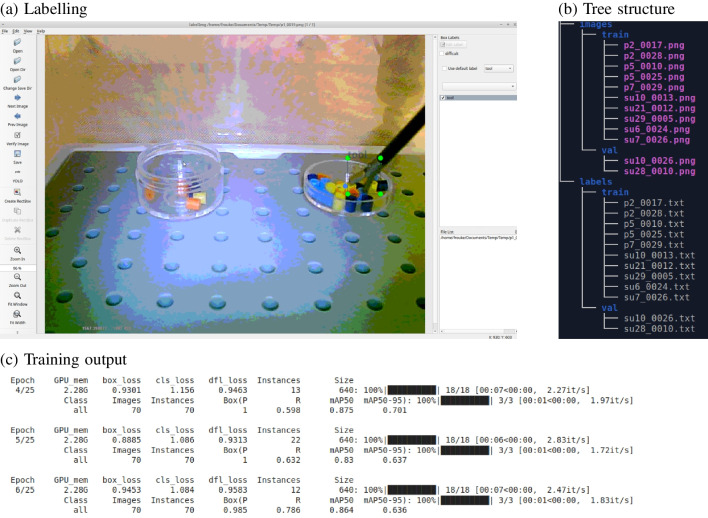
**a** Labelling images with LabelImg. By using the single object and autosave options, labelling can be performed efficiently. To label around 450 images each with a single object, around 45 min was needed. **b** Example tree structure (with a total of 12 images) required for training with YOLOv8 from Ultralytics. **c** Output from the algorithm during training

## Methods

The first set of experiments with YOLOv8 (Jocher et al., 2023) will make use of videos recorded during an eye–hand coordination study with complete novices using a surgical simulator (unpublished data). The aim of this study was to reveal possible signals in the instrument and eye movements that may predict performance in the task. The present study will focus on detecting the position of the instrument in the videos (the tooltip of a surgical grasper^1^).

### Data collection

Eye-tracking data and video recordings were collected from a total of 38 participants (ten males), recruited by word of mouth among students or staff at the University of Aberdeen (UK) with no experience in the use of surgical instruments. All provided written consent for their participation in the study that was approved by the local ethics committee (School of Psychology, University of Aberdeen, UK).

The experimental setup for data collection is shown in Fig. 1. Participants were standing with their head resting in a chin rest (UHCOT Tech Headspot, mounted on a wooden frame) and performed a task with a simulator box (Ethicon endo-surgery inc.) while an EyeLink 1000 system (SR Research, Ontario, Canada) recorded their eye movements and a webcam recorded the inside of the simulator box and projected the image on a Dell 19-inch flatscreen monitor. The task involved a single-use surgical grasper, shown in Fig. 1c, and required participants to move coloured beads from one dish inside the box to another dish (see Fig. 1d) for a total of 3 min. In the present context, only the video recordings are used, not the eye-tracking data.

### Image annotation

A total of 436 images were extracted from a large portion of the videos taking one frame every 10 s. This sampling frequency was chosen such that around 450 images were obtained from the various videos while avoiding images that looked too similar. Using the labelImg software, bounding boxes were drawn around the tooltip in each image, as illustrated in Fig. 2a. Bounding boxes were drawn so that they included all of the metal region of the tool tip. This could sometimes lead to part of the (black) shaft of the instrument to be included in the bounding box. The target object for this analysis is therefore the tooltip, as this is the part of the instrument that participants are expected to fixate during the task. Most images contained the tooltip (386 images), but some images without the tool were also kept to determine whether the model did not detect an instrument when there was none.

LabelImg has the option to automatically save the labels in YOLO-format. If other software is used, it may be required to convert the format of the labels. The YOLO-format requirement for the image in Fig. 2a is shown in Listing 1. The first number (‘0’) indicates the class (which starts counting at zero). The next two numbers indicate the bounding box midpoint (as a proportion of the width and height of the image) and the final two numbers indicate the bounding box width and height (also as a proportion of the width and height of the image). One label file is stored for each image.
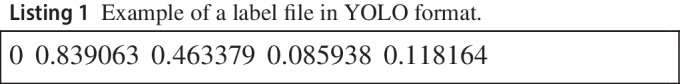


### Model training

During model training, the weights of the pre-trained model are adjusted to better detect the new target object. This training is performed in epochs. In each epoch, the model sees the entire set of images, e.g. 80 images for an annotated set of 100 images. The remaining 20 images (the validation set) are used for determine whether performance was improved during the training step. The model has many parameters (called ‘hyperparameters’), such as the learning rate, the batch size, and the optimiser type. All these parameters were set to their default values.

To perform the training, the set of training images and their annotations were moved into image and label folders and subdivided into training and validation sets, as illustrated in Fig. 2b. A file with instructions (‘yaml’ format) was then created which indicated the label for the single class (‘tool’) and the location of the folders with training and validation images (see Listing 2). The folders and yaml file were then transferred to Google Drive for training on Google Colab.


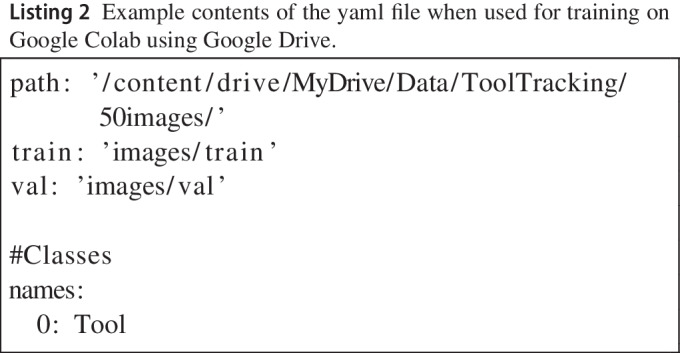


A notebook was created that first installed the Ultralytics package (version 8.0.168), then downloaded the ‘nano’ pre-trained model, and then trained the model for 50 epochs (see Listing 3). In each epoch, the model goes through the entire set of training images, allowing the model to update the weights in the artificial neural network. The results were then transferred to Google Drive using the Shutil package (see Listing 3) and downloaded onto the local computer for inference (prediction) on the entire set of annotated images and two videos not used thus far (See Listing 4 for a minimal example).
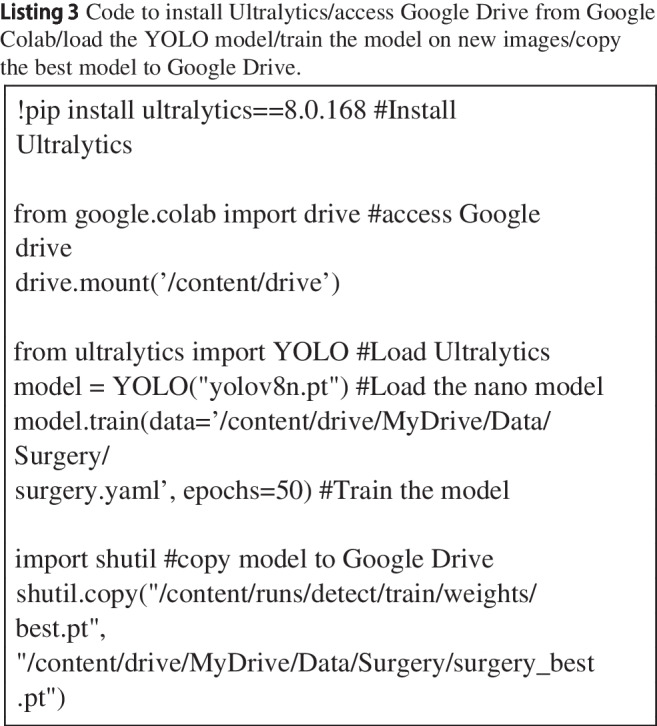

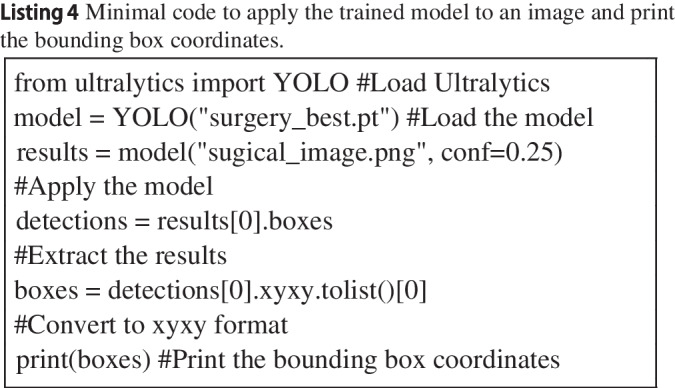


**Fig. 3:**
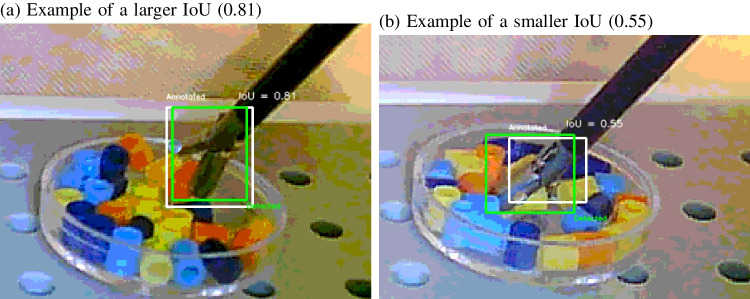
Examples of a larger (IoU = 0.81) and smaller intersection over union (IoU = 0.55). The *white box* shows the annotation and the *green box* shows the detection

### Validation of the models

During training, the Ultralytics package provides two measures of the mean average precision (mAP), the mAP50 and the mAP50:95 (see Fig. 2c). Because there is a single object class (‘tool’), mean average precision is the same as the average precision (AP). When there is a single target object, the mAP50 determines in how many images the overlap between the annotated and detected bounding box is at least 50% (defined as the intersection over union, IoU, see Fig. 3 for examples). The mAP50:95 varies the required overlap between the detected and annotated bounding boxes between 50% and 95% in steps of 5% and computes the average precision over these required overlaps.

The mAP values reported during training are computed on the validation set. These are based on 20% of the annotated images (e.g. 20 images when using 100 images in total). Because the validation set can be small in situations where fewer annotated images are used, the performance on the entire annotated set of 436 images was also compared across image set sizes. Because models trained on larger numbers of images see more of the original 436 images during training, models based on more images may have an advantage on the entire annotated set. Models were therefore also compared on two yet unseen videos. Performance for these videos was determined by counting how often the detected bounding included most of the tool tip, as in the examples in Fig. 6 (corresponding to an IoU over 50%) the number of times the bounding box was clearly not around the tool tip (like in the example in Fig. 9), the number of false positives (a tooltip was detected where there was none) and the number of false negatives (tooltip not detected where there was one).

### Testing the effect of the number of annotated images

To determine how many annotated images are required for adequate object tracking 50, 80, 100, 120, 150, 200, 250, 300, and 350 images were randomly selected from the original image set of images containing a tooltip (only positive examples were used for training; the negative examples were kept for validation after training). Sets of images were split into 80% training and 20% validation images. For example, for the 100 images set, 80 images went into the training set and 20 images into the validation set.

**Fig. 4:**
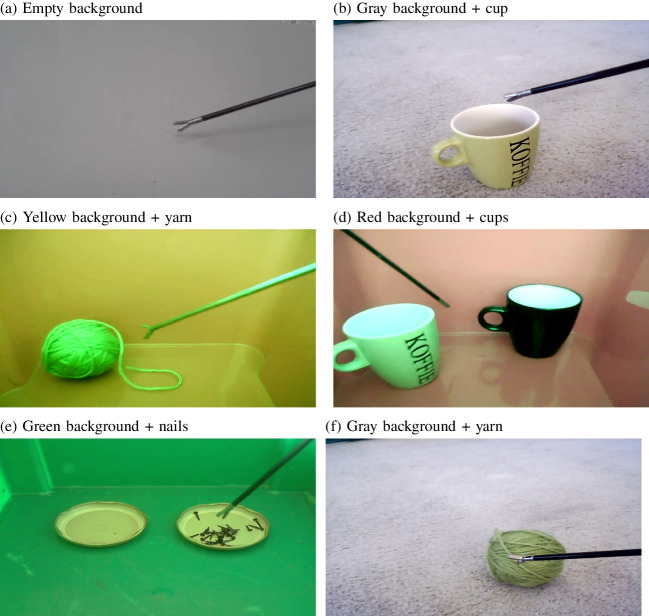
Six different backgrounds that were tested with the original model trained on images inside the surgical simulator. Subsequently, separate models were trained on images for each of the backgrounds, and a model across all backgrounds. Note that the *yellow background* used a slightly different surgical grasper

### Comparing nano, small and medium-sized models

Training typically starts with a pre-trained model, which has been trained on a large dataset. YOLOv8 offers pre-trained models of various sizes: nano, small, medium, large and extra-large. The nano, small and medium-sized models were compared to determine whether performance is affected by the size of the pre-trained models. Since no improvements were found for the small and medium-sized model compared to the nano model, the two larger models (which take substantial time to train and use on images) were not tested. For the same reason, the remainder of the experiments were completed with the nano model.

### Comparing YOLO versions

Within the set of pre-trained models, models could also be found for YOLOv3 and YOLOv5 (besides the YOLOv8 model). To examine whether the YOLO version affects performance, these older models were trained on the 150 and 350 image set sizes, as these showed good performance for the YOLOv8 model. Performance was then compared to that of the YOLOv8 model on both the validation set during training and the entire annotated set.

### Testing the model on other backgrounds

New videos were recorded from the same instrument or a slightly different instrument (exploring the effect of the instrument) in various backgrounds (see Fig. 4). From these videos, images were extracted, which were labelled with the same LabelImg software. The model trained on 150 and 350 images from inside the simulator was then applied to these labelled images and detection performance compared.

### Training different models for different backgrounds

The original model performed poorly on the new backgrounds. New models were therefore trained to determine whether retraining the model would be a feasible strategy when an old model fails to detect an object in a new context. For the ‘empty’ background, this involved 198 images. For the gray background with cup there were 276; for the gray background with yarn there were 240 images; for the green background with nails 356 images were used; for the red background with cups 176 images were available; and for the yellow background with yarn 155 images were annotated (the number of images depended on the lengths of the original videos, as a fixed sampling rate was used).

### Training a single model on various backgrounds

To determine what performance can be achieved across various backgrounds, a new model was trained on all images of all backgrounds (the images of the six different background and the original set of images from inside the simulator, making a total of 1893 images). This model was then applied to the same set of images to assess performance.

### Training a single model on a smaller number of various backgrounds

The model trained on all backgrounds achieved better performance than specific models trained separately for each background. The model trained on all backgrounds, however, was trained with many more images than the model trained on each single background. To examine the extent to which the large number of images affected performance of the model, a new model was trained on a subset of images. From each context, 60 images were selected. To examine whether diverse contexts generalise to an unseen context, no images of the original simulator setting were used for this training. The total number of images was set to 360, comparable with the largest set used for training the original surgical simulator model. The model was then applied to all 1893 images from the various backgrounds.

### Random number generator

As will be shown in the results, performance of the model broke down for image set sizes of 200 and 250 images. This may be an artefact of the random selection of images for the training and validation sets and the subsequent splits into training and validation images. These selections are controlled by the random number generator that can be set by setting the random seed. The original seed of ten was therefore changed to the (also arbitrarily selected) value of 20 and the entire process of creating image set sizes of 50 to 350 images and training and validating the models was repeated with this new setting of the random number generator. If the poor performance persists for 200 and 250 images, this would suggest a different cause for the poor performance for these image set sizes.

**Fig. 5:**
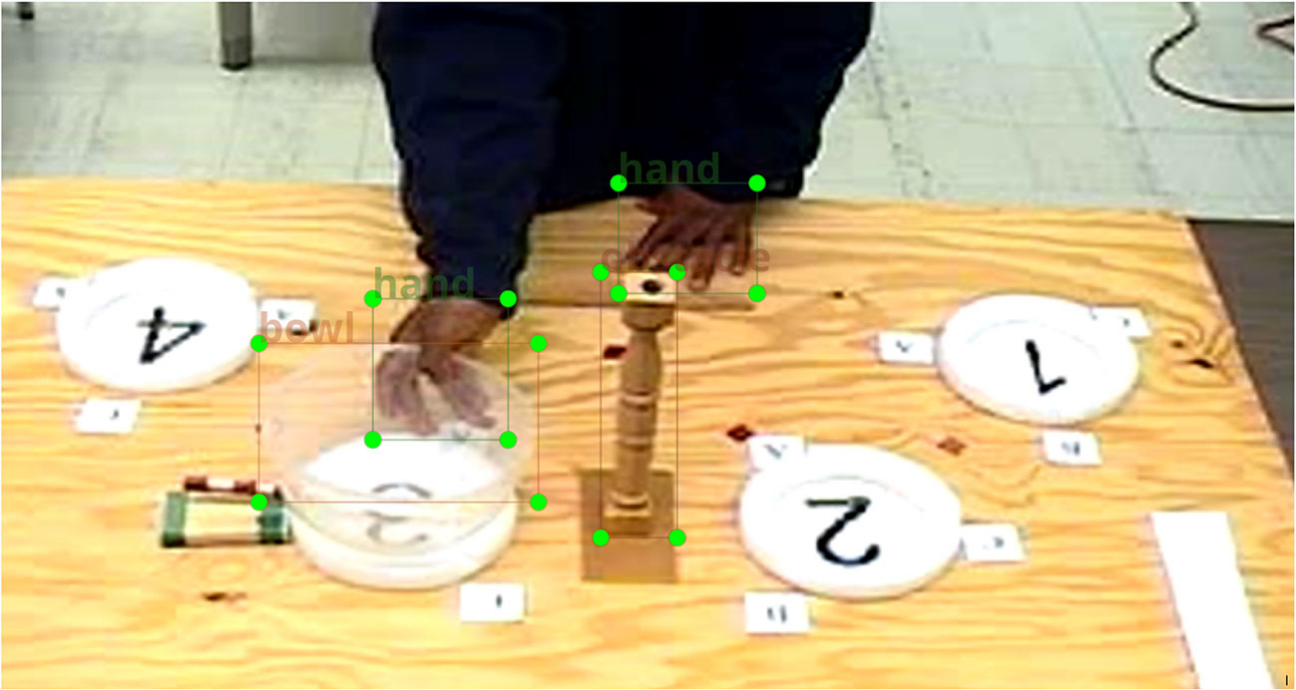
Example image of the bowl transport task in which participants were asked to pick up a transparent salad bowl and to move it to a target ring and gap (for more details, see Hermens et al.  2014). This image shows the end of a movement because the pointer attached to the bowl aligns with the wooden block with two green stripes that indicated the target ring and gap. Participants had to take into account the obstacle that was always placed between the start ring (in this case, ring number 2) and the end ring (in this case ring number 3). Because the position of the hand on the rim is the measure of interest, only the bowl’s rim is annotated, not the entire bowl

**Fig. 6:**
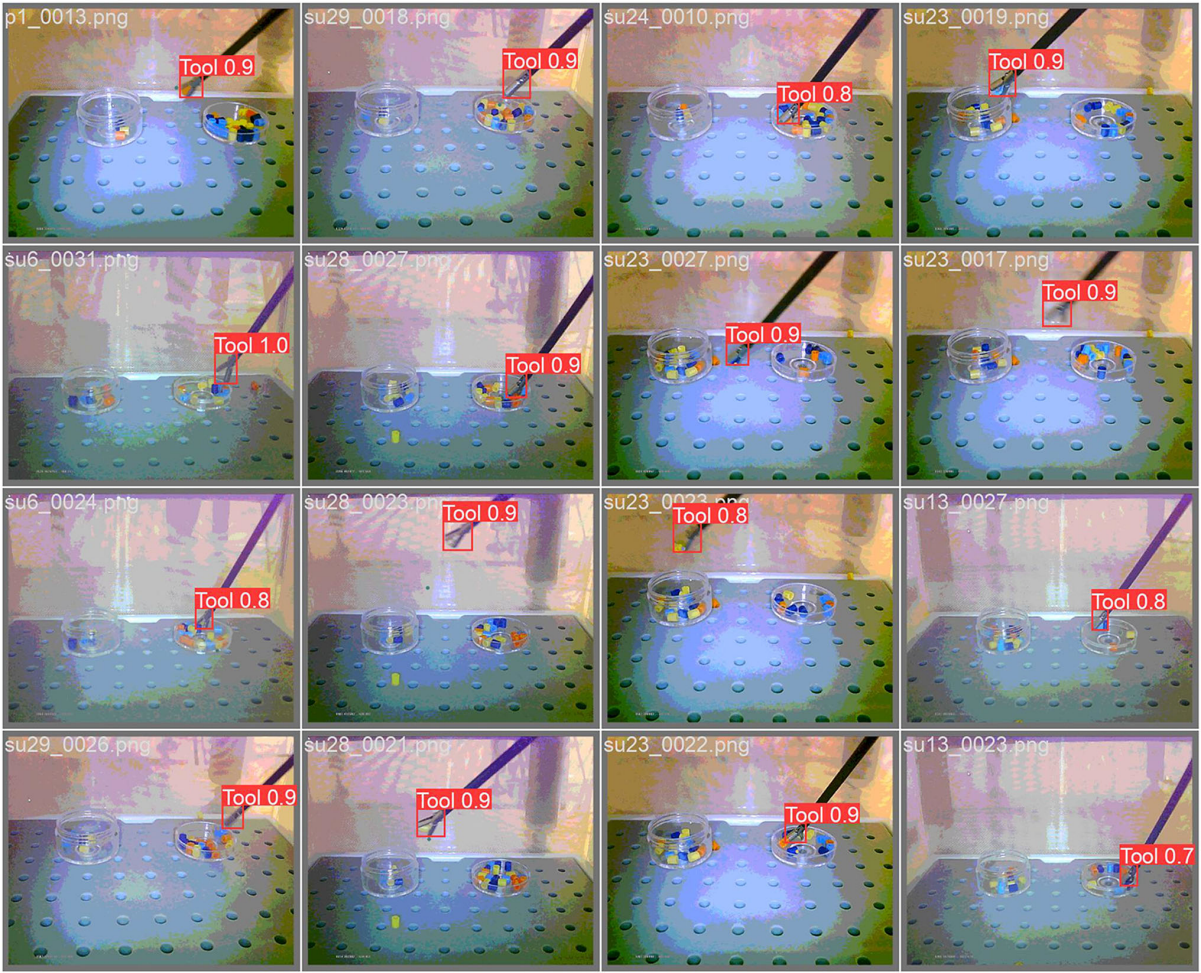
Examples of detections for a model based on 150 annotated images, based on the validation set. The *numbers* shown indicate the confidence of the model (ranging from 0 to 1, with 1 being the highest confidence)

**Fig. 7:**
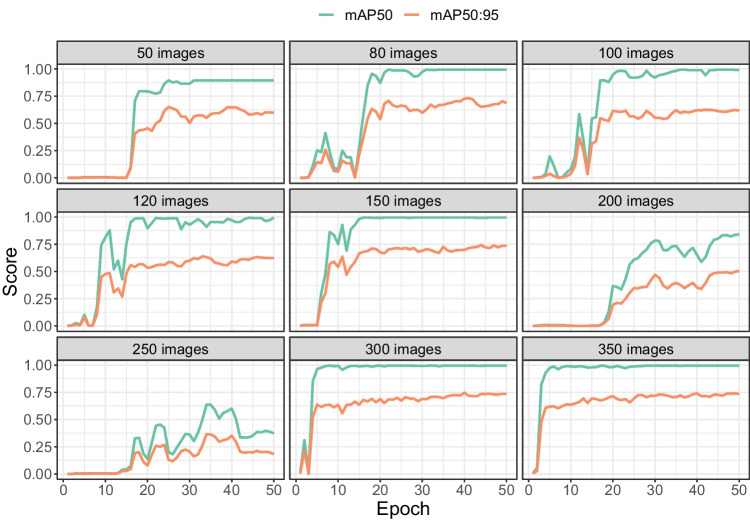
mAP50 and mAP50:95 as a function of the number of images (*subplots*) and the number of epochs (*horizontal axis*). The scores were computed on the 20% validation images during training

**Fig. 8:**
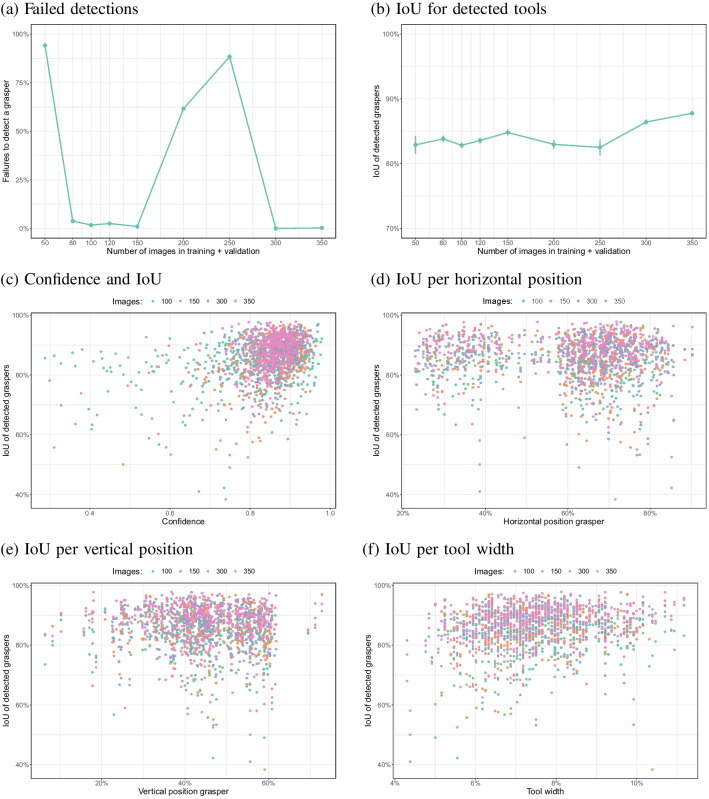
**a** False negatives (failed detections) per image set size. **b** The intersection over unions (IoU) for the detected tools per image set size. **c** IoU as a function of the confidence value. **d** IoU as a function of horizontal position of the tooltip. **e** IoU as a function of the vertical position of the tooltip. **f** IoU as a function of the size of the tooltip. *Error bars* in **d** show the standard error of the mean across images

### Other objects

To examine whether the surgical instrument results extend to other types of objects, object detection was performed on a different dataset. The dataset that was used for this was collected in 2003 as part of a project examining whether participants anticipate their final postures when grasping an object (examining the so-called ‘end-state comfort effect’, Hermens et al., 2014). The published data from this project were analysed using manual annotation.

Two further experiments not included in the original publication involved participants moving a salad bowl around an obstacle. The measure of interest in these experiments was where on the rim of the bowl participants take hold (expressed as an angle). To determine this measure, the midpoint of the bowl’s rim has to be determined, as well as the position of the hand. For the current analysis, the position of the obstacle will also be annotated, because it will allow determining which way participants moved around the obstacle, and provides a baseline object that is not transparent (bowl) and does not change shape (hands).

The reasons for using this dataset are the following. First, because in 2003 data storage was expensive, the videos were compressed to an MPEG-1 format with a frame resolution of 880 by 540 pixels. This dataset will therefore demonstrate whether the results extend to situations with a relatively low image quality. Second, the transparent bowl could pose problems to the object detection algorithm, because its texture and brightness is expected to depend on the background. The comparison with the obstacle with show whether transparency of the object plays a role. Third, hands differ in shape and colour, which may pose another problem for the object detection algorithm. Finally, in many of the images there is some form of occlusion of the objects, which may form another challenge to object detection.

The setup and example annotations of the various objects of interest are shown in Fig. 5. In this example, all three objects of interest (bowl, hands, obstacle) were annotated, but for object detection, each object was annotated separately and models were also trained separately^2^. A total of 450 images were extracted from 14 videos at intervals of around 10 s. Using the same seed of the random number generator as for the original analyses for the surgical instrument (seed = 10), these images and annotations were split into sets of sizes 50, 80, 100, 120, 150, 200, 250, 300, and 350 images, only using images that contained the target object (bowl, hand, or obstacle). For each set size and each object a YOLOv8 model was trained and the validation results collected.

### Statistical comparisons

The validation process during training yields a mAP-50 and a mAP-50:95 score, which is a single number per epoch, computed across all images. These values can therefore only be compared statistically when repeated selections of images from the original dataset and repeated splits between training and test sets are performed and the model is retrained for each new selection and split. This is computationally expensive. Moreover, these values were often based on a small dataset (e.g. 20 images when 100 annotated images were used), meaning that the mAP values may be variable.

Instead, statistical comparisons were performed on failed detection rates and IoU values when the models were fitted on the entire set of images. Unless specified otherwise in the text, mixed effects models were fitted with and without the fixed factor of interest (e.g. the image set size) with image as a random factor, which takes into account related datapoints for the same images. Models were compared with a likelihood ratio test yielding a $\chi ^2$ value and a *p* value indicating whether the nested model without the fixed effect of interest fitted the observations significantly worse than the model with the fixed effect. For this analysis the *lme4* package in *R* was used.

## Results

Generally, good detection of the tooltip was obtained for many of the image set sizes. Figure 6 shows examples of detections for the model trained on 150 images, showing that the tooltip was detected in every single of this random selection of images from the validation set.

### Validation scores per image set size

Figure 7 shows that mAP50 and mAP50:95 scores improved with more training epochs, but leveled off at some point. There were, however differences between image set sizes. Lower numbers of images (50, 80, 100, 120, 150) all converged to almost perfect mAP50 scores (i.e. the approximate location of the tooltip is detected with high consistency). mAP50 values were lower for image set sizes of 200 and 250, but returned to almost 100% when the number of images was further increased to 300 and 350. To reach the near perfect levels of mAP50, fewer training epochs were needed with 300 and 350 images than with smaller numbers of images (80, 100, 120, and 150).

For the more stringent mAP50:95, none of the image set sizes yielded a performance over 80%. For this measure, a better overlap between the detected and annotated bounding box is required to achieve high scores. This implies that for very accurate localisation of the tooltip, rather than just detection, more images may be needed (or a different approach may be required, such as pose estimation, see the Discussion).

### Detection accuracy on the entire annotated set of images

To validate each of the models on the entire set of annotated images (40 images without a surgical instrument and 396 images with a surgical instrument), the model frozen at the epoch with the best mAP50 value was used. A confidence threshold of 0.25 was applied for detection. In instances where multiple detections were made by the model, the overlap values were pooled across detections.

False positives (i.e. a detection of a tool when there was none, across 40 images) did not occur for any of the models. False negatives did occur. Figure 8a shows that for some image set sizes, the model failed to detect the instrument in a large portion of the images that contained the instrument. Image set sizes of 80, 100, 120, 150, 300 and 350 images led to adequate detection of a tool when there was one (almost zero failures). A significant effect of image set size on false negatives was found ($\chi ^2$(1) = 56.2, $p<$ 0.0001). Significant differences in the false detection rates between subsequent numbers of images were found between 50 and 80 ($\chi ^2$(1) = 977.2, $p<$ 0.0001), 80 and 100 ($\chi ^2$(1) = 11.8, $p<$ 0.0001), 120 and 150 ($\chi ^2$(1) = 17.8, $p<$ 0.0001), 150 and 200 ($\chi ^2$(1) = 412.5, $p<$ 0.0001), 200 and 250 ($\chi ^2$(1) = 83.3, $p<$ 0.0001) and between 250 and 300 images ($\chi ^2$(1) = 941.1, $p<$ 0.0001).

### Overlap between annotated and detected bounding boxes

To examine how well the bounding box fitted the annotated bounding box when a detection was made, the intersection over union (IoU) was computed. This measure ranges from zero (no overlap) to 100% (perfect overlap between the detected and annotated box, see Fig. 3 for examples).

Figure 8b shows that when the tool was detected, there was a high degree of overlap between the detected bounding box and the annotated box of around 80% to 90% with relatively little variation between images (small standard errors of the mean). There was a statistically significant effect of the number of images used for training on this overlap ($$\chi ^2$$(8) = 314.5, $$p<$$ 0.0001). Comparisons between subsequent image set sizes using Bonferroni correction for multiple comparisons indicated a significant difference in the IoU between 80 and 100 images ($$\chi ^2$$(1) = 11.8, $$p<$$ 0.0001), between 120 and 150 images ($$\chi ^2$$(1) = 15.1, $$p = $$ 0.0001), between 150 and 200 images ($$\chi ^2$$(1) = 12.9, $$p =$$ 0.0003), between 250 and 300 images ($$\chi ^2$$(1) = 20.0, $$p<$$ 0.0001) and between 300 and 350 images ($$\chi ^2$$(1) = 19.9, $$p<$$ 0.0001).

**Fig. 9:**
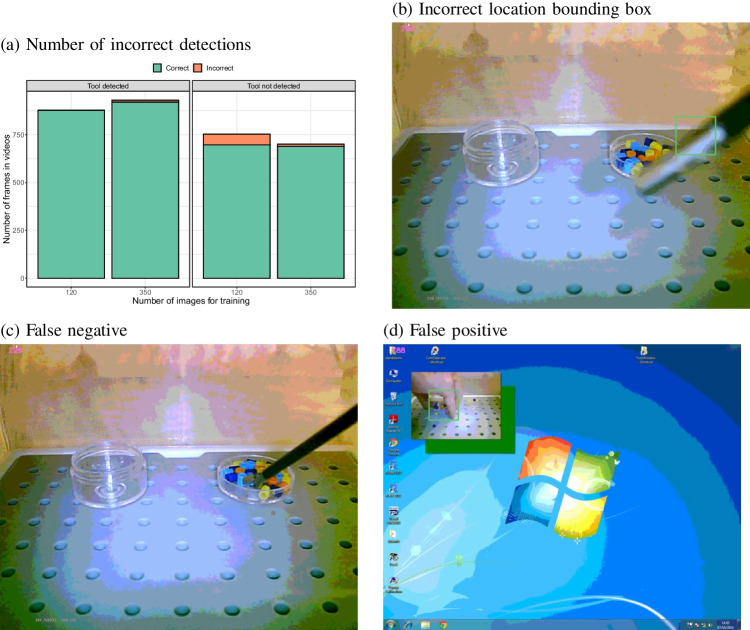
**a** Numbers of incorrect tool detections in two complete videos that unseen during training for two of the models (based on 120 and 350 images). Because no bounding box annotations are available for these videos, the data focuses on false positives and false negatives. **b**–**d** Examples of the infrequent errors by the model trained on 120 images

Statistically significantly higher IoU values were found when the model was trained on more images, but the combination of Fig. 8a and b shows that the main problem of poorly performing models was the lack of a detection of the instrument. Interpolation techniques (i.e. inferring the tool position from previous or subsequent video frames) may be used to recover the instrument’s position for frames where detection fails when there are few false negatives.

The model provides a confidence score with each bounding box, which indicates how certain the model is that the bounding box contains the target object and how large the overlap (IoU) with the target box is. Figure 8c plots the agreement between confidence and IoU for well-performing models. A significant correlation was found across image set sizes (Pearson’s *r* = 0.34, $p<$ 0.0001) and within each of the image set sizes in the plot ($p<$ 0.0001). In most cases, high confidence coincides with a high IoU. There were, however, instances of a lower confidence with a fairly high IoU. The reverse seems to be less common.

Figure 8d and e show that the IoU did not depend strongly on the horizontal or vertical position of the tool on the screen. There were small, but statistically significant negative correlations between horizontal position and IoU (*r* = -0.051, *p* = 0.043, *N* = 1584) and vertical position and IoU (*r* = -0.068, *p* = 0.0073, *N* = 1584) when the results for 100, 150, 300 and 350 images were combined. None of the individual correlations for 100 (x: *p* = 0.084, y: *p* = 0.16), 150 (x: *p* = 0.12, y: *p* = 0.25), 300 (x: *p* = 0.82, y: *p* = 0.099) and 350 (x: *p* = 0.38, y: *p* = 0.18) were statistically significant. There were no clear areas for which the tool’s position was detected less reliably (for the models that detected the tool in almost every frame). Lower IoU values were somewhat more often found for higher positions of the instrument in the image. Figure 8f indicates that IoU values were somewhat lower when the instrument was viewed at a larger distance inside the box (as reflected by a smaller size in the image). This was reflected by statistically significant negative correlations between box width and IoU across 100, 150, 300, and 350 images (*r* = 0.20, $p<$ 0.0001, *N* = 1584) and significant correlations within 100 (*r* = 0.20, $p<$ 0.001), 150 (*r* = 0.17, $p<$ 0.001), 300 (*r* = 0.19, $p<$ 0.001) and 300 images (*r* = 0.30, $p<$ 0.001).

### Detection accuracy for unseen videos

As an additional validation, two of the best performing models (trained on 120 and 350 images) were applied on frames of two complete videos. For each video frame, the detected bounding box was superimposed on the image. The resulting images were then sorted into folders of images with the correct and incorrect detections based upon visual inspection.

Figure 9a shows the counts of correct and incorrect bounding boxes, indicating that the models were correct in most instances. The largest number of errors was made by the model trained with 120 annotated images. These errors were mostly false negatives (a failure to detect the instrument). Examples of the few incorrect detections are shown in Fig. 9b–d, illustrating an incorrect bounding box, a failed detection, and an erroneous detection.

**Fig. 10:**
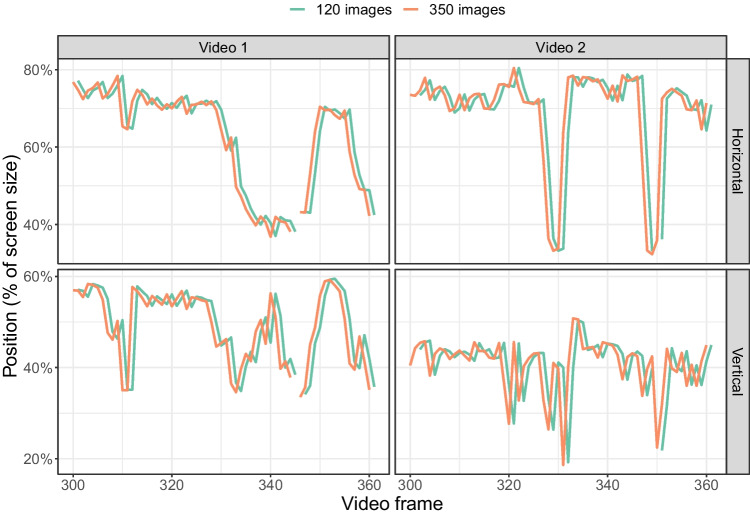
Horizontal and vertical centre of the detected instrument across several video frames. The trace for the 120 images is shifted by one frame to facilitate the comparison between the model trained on 120 and on 350 images

Figure 10 shows the detected horizontal and vertical position of the instrument over video frames for a section of both videos. The two models show very similar detected instrument positions. The correlation between the horizontal positions of the bounding boxes of the two models was almost perfect (first video: *r* = 0.9998, $p<$0.001, second video: *r* = 0.9995, $p<$ 0.001). Similarly high correlations were found for the vertical positions of the bounding boxes (first video: *r* = 0.9991, $p<$0.001, second video: *r* = 0.9988, $p<$ 0.001).

**Fig. 11:**
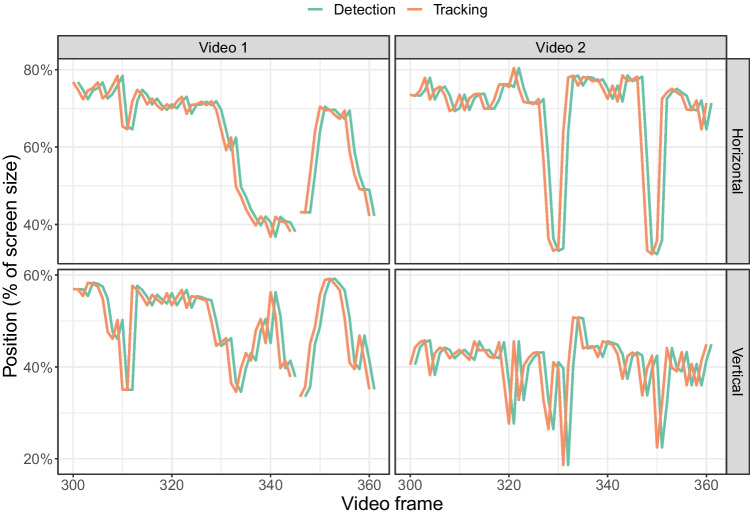
Horizontal and vertical centre of the detected instrument across several video frames, comparing detection and tracking. The trace for the detection data is shifted by one frame to facilitate the comparison between the models. Only the data for the model trained on 350 images are shown

The traces in Fig. 10 were obtained by using the detection model, which does not take into account previous or subsequent frames. YOLOv8 also has the option to perform tracking (using the BoT-SORT or ByteTrack algorithms). Figure 11 compares the midpoint of the detected bounding box between detection and tracking (with the default BoT-SORT algorithm) for the same section of the video, showing that there was little difference between the two methods. There were two video frames for which no object was detected with detection, while an object was found with tracking. The absolute difference in the horizontal position between tracking and detection was close to zero (a value less than 0.000001) and the correlation between the tracked and detected horizontal position was equal to 1 for both videos ($p<$ 0.0001). This indicates that the main difference between the tracker and the detector is that the tracker adds an identifier for each object.

**Fig. 12:**
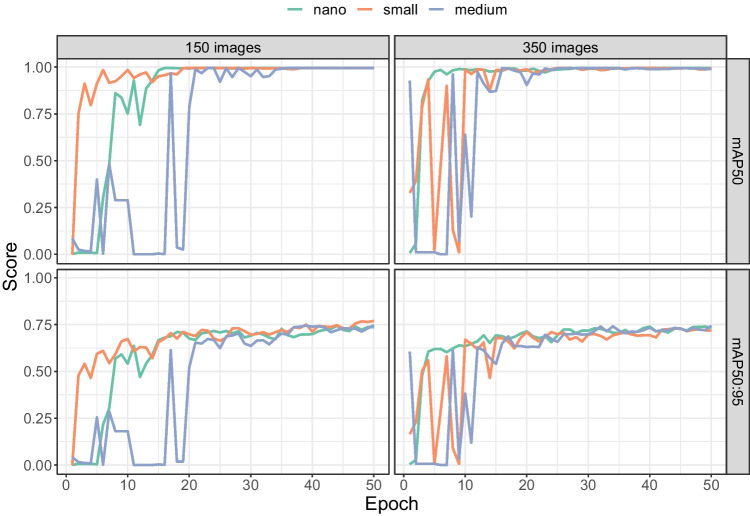
mAP50 and mAP50:95 for models trained with the nano, small and medium pre-trained networks. The mAP scores are computed on the corresponding validation sets for each number of overall images (i.e. 30 images for the set of 150 images and 70 images for the set of 350 images)

### Detection accuracy for different pre-trained model sizes

When training a model for the detection of a specific object, one typically starts with a pre-trained network, so that processing of low-level features such as edges and corners does not have to be trained from the images. Ultralytics offers pre-trained models of various sizes: Nano, small, medium, large and extra-large. So far, the nano model was used as the pre-trained model. The advantage of the nano model is that it trains quickly and is fast when used for processing images and videos (inference), even when used on a PC without a graphical processing unit.

mAP50:95 values on the validation set never reached 100% when trained with the nano model. To examine whether this is a limitation of the nano model, the small and medium pre-trained models were used as the start of training, before moving to larger models depending on whether improvement was found for these smaller models. A smaller (150 images) and a larger (350 images) image set size was used during training.

Figure 12 shows the development of the mAP scores over the training epochs when starting with the nano, small or medium pre-trained model. All models converged to similar mAP scores during training.

**Fig. 13:**
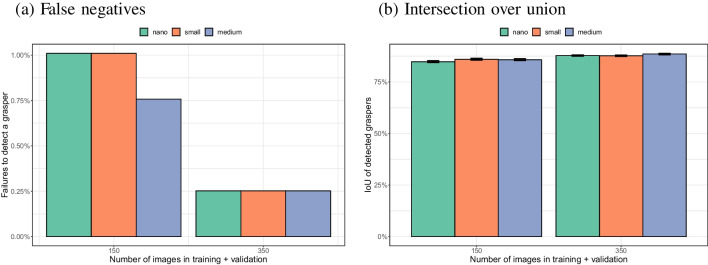
Comparison of the performance of the nano, small and medium models. Smaller models take less time to train and less time to use on new images than larger models. In terms of accuracy of the detections, there is little difference between the three sizes of models

There were no false positives for any of the models (nano, small, medium; trained with 150 or 350 images). Figure 13a shows that false negatives (failures to detect the instrument when present) were infrequent (less than 1%) and occurred less often when more images were used for training. There was no significant interaction between the number of images and the size of the pre-trained model on the number of false negatives ($\chi ^2$(2) < 0.0001, $p>$ 0.99). There was a significant main effect of the number of images on the false negative rate ($\chi ^2$(1) = 25.7, $p<$ 0.001). The main effect of the size of the pre-trained model was not statistically significant ($\chi ^2$(2) = 1.74, *p* = 0.42).

Figure 13b shows that the overlap between the annotated and the detected bounding box improved slightly when a larger pre-trained model was used. There was a significant interaction between number of training images and pre-trained model size on IoU ($\chi ^2$(2) = 8.17, *p* = 0.017). Both within 150 images ($\chi ^2$(2) = 23.0, *p* = 0.000010) and 350 images ($\chi ^2$(2) = 16.5, *p* = 0.00026), there was a significant effect of the pre-trained model size on IoU. Within 150 images there was a significant difference between the nano and the small model ($\chi ^2$(1) = 19.3, *p* = 0.000011), but not between the small and medium model ($\chi ^2$(1) = 0.95, *p* = 0.33). Within the 350 images there was no significant difference between the nano and small model ($\chi ^2$(1) = 0.1955, *p* = 0.66), but there was a significant difference between the small and medium model ($\chi ^2$(1) = 14.4, *p* = 0.00014). The differences in IoU between the various pre-trained models were small (in the order of 1%). The extra training time and stronger demands on computational resources for inference for larger pre-trained models may therefore not pay off.

**Fig. 14:**
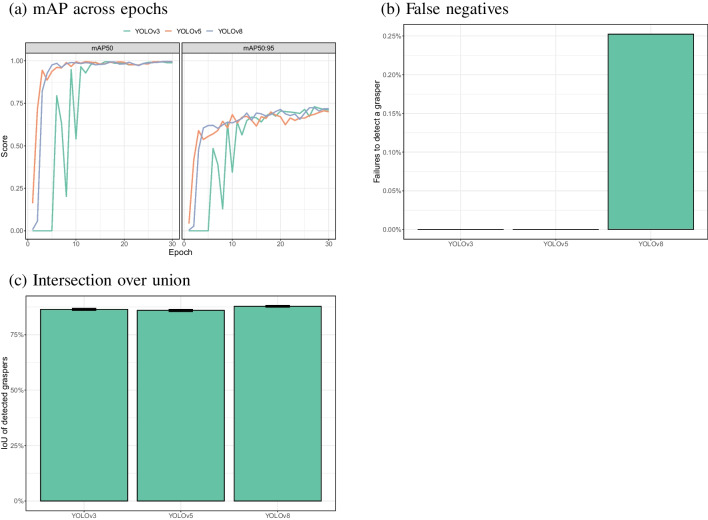
Comparisons of the performance of YOLOv3 (‘yolov3u’), YOLOv5 (‘yolov5nu’) and YOLOv8 trained on 350 images. **a** mAP50 and mAP50:95 as a function of epoch (each training cycle in which the model sees all images in the training set), based on the validation set (70 images in this case). **b**, **c** False negatives and IoU values based on 396 images in which an instrument was present

### Earlier YOLO versions

Figure 14 shows that earlier versions of YOLO (versions 3 and 5) resulted in similar performance as the most recent version when trained on 350 images and over sufficient epochs. There was one false negative for YOLOv8, where there were none for versions 3 and 5. At the same time there was also a statistically significantly higher overlap between the annotated and detected bounding boxes for YOLOv8 (IoU; compared to version 3: $\chi ^2$(1) = 21.7, $p<$ 0.001, compared to version 5: $\chi ^2$(1) = 43.1, $p<$ 0.001). YOLOv8 took less time to train and use when trained than the other two models^3^ and the final model was also substantially smaller in size.

### Different backgrounds

**Fig. 15:**
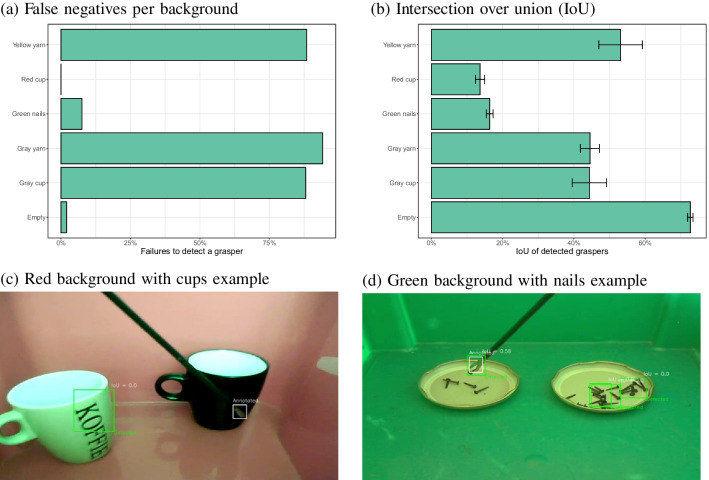
**a**-**b** False negatives and IoU for detected instruments for each of the other backgrounds for a model trained on 350 images inside the surgical simulator box. **c**–**d** Examples of incorrect detections for two of the backgrounds

When the model trained on 350 images from inside the surgical simulator was applied to the images from the six other backgrounds, no false positives were observed (a tool detected when there was none, across 190 images without a tool, with a confidence threshold of 0.25). Figure 15a shows that the original model failed to detect the tool in many of the images with other backgrounds. Lower false negative rates were found for the red, green and empty backgrounds. Compared to the best performing background (‘red cup’), significantly higher false negative rates were found for the gray cup ($\chi ^2$(1) = 430.5, $p<$ 0.001), the gray yarn ($\chi ^2$(1) = 469.7, $p<$ 0.001) and the yellow yarn ($\chi ^2$(1) = 361.5, $p<$ 0.001), but not for the green nails ($\chi ^2$(1) = 1.59, *p* = 0.21) or the empty background ($\chi ^2$(1) = 0.23, *p* = 0.63).

**Fig. 16:**
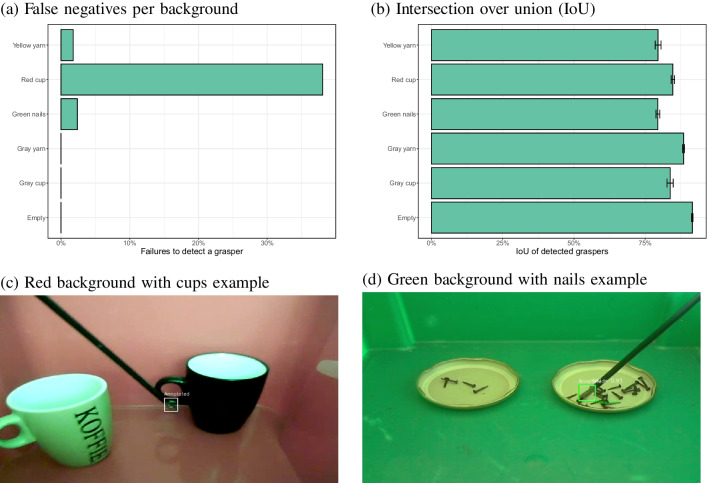
**a**-**b** False negatives and IoUs for detected instruments for each of the other backgrounds, for a model trained on the specific images with this background. **c** Example where the model fails to detect the instrument. **d** Example of a successful detection in the presence of nails

Detections were, however, often away from the actual position of the tool, as shown by the low IoU values in Fig. 15b. Only the empty background showed a combination of a high IoU and a low false negative rate. Compared to this empty background, significant differences in IoU were found for the red cup ($\chi ^2$(1) = 554.3, $p<$ 0.001), green nails ($\chi ^2$(1) = 609.4, $p<$ 0.001), yellow yarn ($\chi ^2$(1) = 36.2, $p<$ 0.001), gray yarn ($\chi ^2$(1) = 75.4, $p<$ 0.001) and the gray cup ($\chi ^2$(1) = 89.6, $p<$ 0.001). For the red background the letters on the cup often led to incorrect detections (Fig. 15c). For the green background, the nails often caused to false positives (Fig. 15d). While the tooltip was often detected, it will be difficult to tease these detections apart from detections of the nails. The average IoU was computed over all detections, which explains the relatively low average values (there were many cases of zero overlap when a nail was detected as the tooltip).

**Fig. 17:**
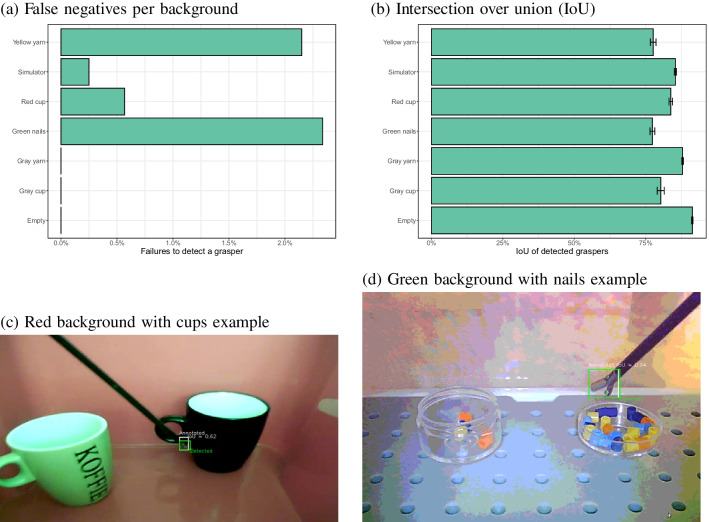
(**a**–**b**) False negatives and IoUs for detected instruments for each of the other backgrounds, for a model trained on images from all backgrounds (including the original simulator box). Note that the scale for (**a**) is adjusted to the low rates of false negatives. **c** Good detection for the red background. **d** Good detection for the original setting

**Fig. 18:**
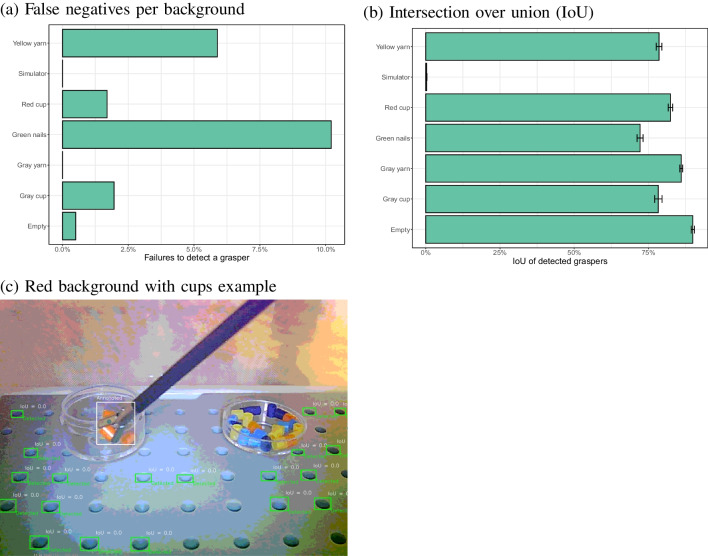
**a**–**b** False negatives and IoUs for detected instruments for each of the backgrounds, for a model trained on 60 images from each background, except the simulator background. **c** For the simulator background, which was not used for training, the holes in the simulator floor were detected, but not the instrument itself

**Fig. 19:**
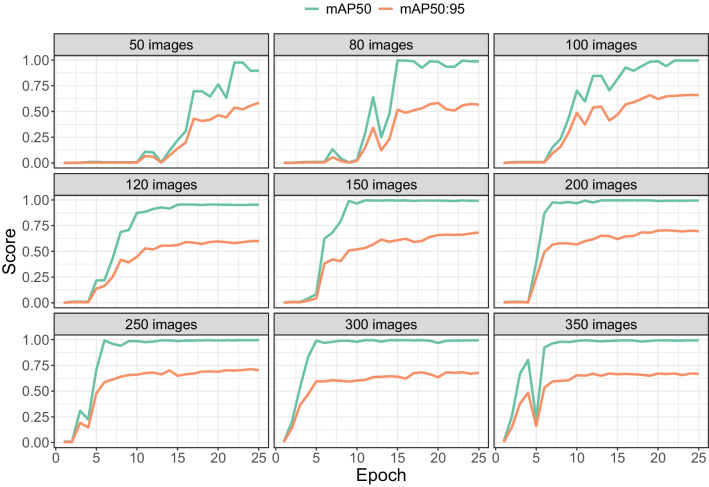
mAP values for detecting the surgical instrument with a different setting of the random number generator for selecting the images for the different image set sizes and splitting the images between training and validation

**Fig. 20:**
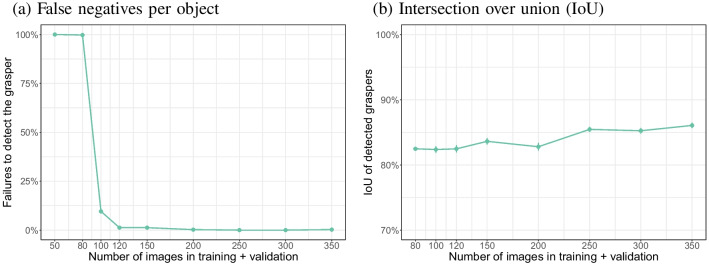
False negatives and IoUs for detected instruments per number of images in the combined training and validation sets for a different setting of the random number generator

**Fig. 21:**
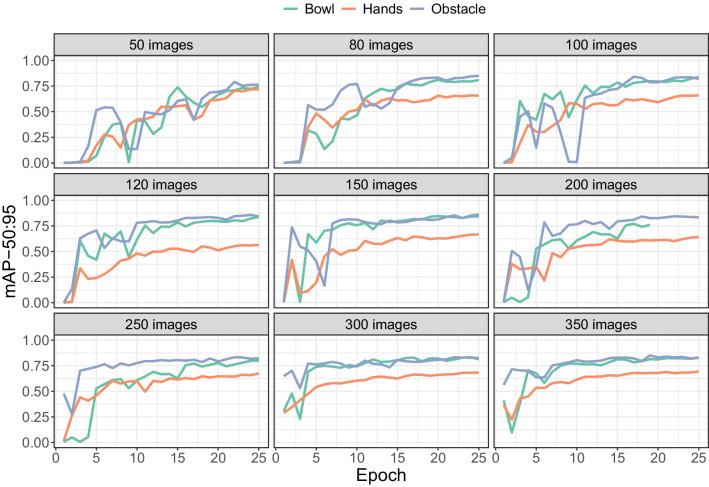
mAP50:95 values for the three objects from the bowl grasping data. As for the surgical tool, mAP50 rates approached 1 (data not shown), but most mAP50:95 scores remained below 0.8

**Fig. 22:**
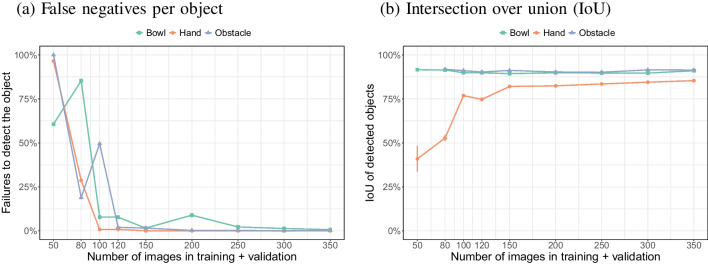
False negatives and IoUs for detected objects per number of images in the combined training and validation sets

### Training new backgrounds separately

To examine whether the problems with detections for other backgrounds than the surgical simulator could be solved by training specific models for each of the backgrounds, new YOLOv8 detectors were trained on each of the subsets of images (one model per background). All of the annotated images were used, except the ones that did not contain the instrument.

Figure 16 shows the results when the specific models were applied to the images that it was trained on, plus the images not containing the instrument. First of all, there were no false positives (no instrument was detected when there was none). Figure 16a shows that false negatives were infrequent, except for the red background with the cup. Significantly higher failed detection rates were found for the red cup than for the green nails ($\chi ^2$(1) = 83.2, $p<$ 0.0001) and the yellow yarn ($\chi ^2$(1) = 49.2, $p<$ 0.0001). Figure 16c shows that these false negatives may be due to a low contrast between the instrument shaft and the cup. Figure 16b shows that there was a good, but not perfect overlap between the annotated and detected bounding boxes for all backgrounds. Compared to the empty background, all other contexts performed statistically significantly worse (all *p* values < 0.001). Figure 16d shows that after training the model specifically on the green background images, the problem with the detections of nails no longer occurred.

### Training all backgrounds together

One solution to the problem of a model failing with new backgrounds proved to be to train a new model for that specific background. If the background is not known in advance, this may not be a feasible strategy. It was therefore examined whether a single model, trained on all backgrounds, performs similarly well on images from all backgrounds.

Figure 17a shows a lower false negative rate for the red cup context ($\chi ^2$(1) = 99.4, $p<$ 0.001) with a model trained on all backgrounds, compared to models trained on each background separately (no significant differences were found for the other contexts). No false positives were observed. Figure 17b shows that the IoU was high for all backgrounds. No significant improvement in the IoU was found between the separate models for each context and the combined model across all contexts after Bonferroni correction (all uncorrected *p* values > 0.023). Figure 17c and d show two examples of a good detection with the model trained on all backgrounds.

### Subset of diverse backgrounds

To test whether good performance was maintained when fewer training images of various backgrounds are used during training, a model was trained on a subset of 360 images, 60 from each background, except from inside the surgical simulator. Figure 18 shows that this model performed fairly well on all backgrounds, except for the surgical simulator box that was not used during training. There were significantly more false negatives compared to when 1893 images were used for training for all contexts (all *p* values < 0.0011), except the red cups ($\chi ^2$(1) = 1.05, *p* = 0.31), but the difference in rates was small. IoU values for the detections were highly similar to those of the model trained on many more images (except for the simulator environment that was not used for training). Significant differences in IoU were found for the green nails ($\chi ^2$(1) = 27.2, $p<$ 0.0001) and the simulator environment ($\chi ^2$(1) = 20163, $p<$ 0.0001). This means that when expecting a diverse background all backgrounds need to be used for training the model, but large numbers of images are not strictly needed.

### Random number generator

Figure 19 shows the mAP50 and mAP50-95 as a function of the number of images in the combined training and validation set and the number of epochs for the different setting of the random number generator. The curves for the 350 images show a dip at around five epochs but continue towards a maximum value afterwards. In contrast to the previous setting of the random number generator (Fig. 8), all curves show an increasing trend, with more training epochs needed for smaller image set sizes.

When applied to the full set of annotated images, large numbers of false negatives are now only seen for image set sizes of 50 and 80 (Fig. 20a). False detection rates significantly differed between image set sizes ($\chi ^2$(1) = 3262.4, $p<$ 0.0001). A significantly higher false negative rate was found for 100 than for 120 images ($\chi ^2$(1) = 131.8, $p<$ 0.0001), but not between 120 and 150 images ($\chi ^2$(1) < 0.001, *p* = 0.999). Significantly lower false negative rates were found for the seed equal to 10 for 50, 80, and 100 images ($p<$ 0.0001). Significantly higher false negative rates were found for the seed equal to 10 for 200 and 250 images ($p<$ 0.0001). For 120, 150, 300 and 350 images, there was no significant difference in the false negative rates between the seeds (smallest uncorrected *p* value = 0.047).

When the instrument was detected, the intersection over union (IoU) was around 85%. There was a statistically significant effect of number of images on the IoU ($\chi ^2$(1) = 124.6, $p<$ 0.0001). A significant difference between subsequent numbers of images was found between 120 and 150 images ($\chi ^2$(1) = 6.43, *p* = 0.011) and between 200 and 250 images ($\chi ^2$(1) = 15.8, $p<$ 0.0001), but not between other subsequent numbers of images. The IoU values for the seed equal to 20 were similar to those for the seed equal to 10, but significantly different IoU values were found for 300 images ($\chi ^2$(1) = 7.92, *p* = 0.005) and 350 images ($\chi ^2$(1) = 20.6 $p<$ 0.001). Together these results indicate that the poor performance for the image set sizes of 200 and 250 previously were due to the random assignment of images to the training and validation sets of various sizes.

### Other objects

Figure 21 shows the mAP50:90 rates for the three objects from the bowl grasping data. Whereas the mAP50 rates approached 1 for all three objects (data not shown), mAP50:95 rates stayed below 0.80 in almost all cases. This means that even for a rigid object like the obstacle, the model was struggling to fit a tight bounding box. These validation rates do not tell the full story, because example validation images suggest that for smaller sets of training images, the model often failed to detect the object (data not shown).

Figure 22 shows that false negative rates (failed detections) drop quickly for all three objects when increasing the image set size to 100. There was a significant interaction between type of object and number of training images on the false negative rate ($\chi ^2$(2) = 1120, $p<$ 0.0001). Significantly different false negative rates were found for each of the number of images (*p* values all < 0.0001), except for 350 images (uncorrected *p* value = 0.025).

The intersection over union (IoU) for detected objects was around 80% for bowls and obstacles and was relatively constant over the image set size (no data point is shown for 50 images for the obstacle, because no obstacles were detected for this image set size). The IoU for detected hands increased with the image set size to a value lower than that for the bowl and obstacle. These observations were reflected in a significant interaction between object and number of images ($\chi ^2$(2) = 485.1, $p<$ 0.0001). For all numbers of images, there were statistically significant differences in the IoU between the three objects (all *p* values < 0.0001). The IoU for the obstacle and the bowl was only significantly different for 150 images ($\chi ^2$(1) = 13.4, *p* = 0.0002) and 300 images ($\chi ^2$(1) = 13.5, *p* = 0.00024). The IoU for the hands was always significantly lower than for the bowl or obstacle (all *p* values < 0.0001). The lower IoU for the hands may have been due to the more variable shape of the hands compared to bowl and obstacle.

Together, these results suggest that 100 to 150 images could be enough to train a model for the various objects (and that detection is excellent, but localisation a bit worse). To achieve more stable results image set sizes of 300 or 350 images are recommended.

## Discussion

The present study examined the use of YOLOv8 (Jocher et al., 2023) to detect objects in images and videos. A typical lab setting was chosen, where the same object is used within the same context, recorded with the same camera and under the same lighting conditions. Annotation of images takes time and it is therefore important to know how many images are needed to train an object detector. The present results suggest around 120 to 150 images suffice, but for reliable performance it is better to annotate 300 to 350 images. Similar effects of the number of training images on detection performance were also found for images of a bowl grasping task. These results are in line with those by Li et al. (2018) who used older versions of YOLO for playing card detection. Good detection of surgical instruments is also in line with earlier work with older YOLO versions (Choi et al., 2017, 2021; Wang et al., 2021) who typically used substantially larger numbers of images (e.g. 1344 images of a grasper in Wang et al., 2021).

The number of images available for training may depend on how much video material is available to extract video frames from. In the present study images were extracted at intervals of around 10 s. If less video material is available a higher sampling rate may be needed, which may also affect the similarity of the images (depending on how much the scene changes over time). Future studies should look closer into the role of sampling rate and image similarity, but this may require extensive computing. As a rule of thumb, researchers could start by annotating around 350 images and increase this number if poor object detection is obtained.

Interestingly, for the initial simulation with the surgical simulator box, performance dropped for intermediate numbers of images, contrasting the findings by Li et al. (2018) who found a monotonically increasing relation between performance and image set size. A second experiment was therefore performed in which the setting of the random number generator was changed. This led to a different selection of images for each image set size, and different splits between training and validation sets. This showed that the drop for intermediate numbers of images was an anomaly associated with the sampling of the images. This was reinforced by the results for the bowl study data, which also showed increasing performance with increasing numbers of images.

More training epochs were needed when fewer images were used (around 20) than for many images (fewer than ten epochs). Li et al. (2018) suggest that a large number of epochs could lead to overlearning (poor generalisation to unseen images), but this did not occur until 1000 epochs in their study. Training with YOLOv3, YOLOv5 and YOLOv8 for the present data already occurred within 30 epochs and there were no signs of poor performance on unseen images. The present data suggest that annotating additional images may be the best use of time when the object detector does not yet perform well, because it also means a reduction in the number of epochs needed for training.

While detection of the approximate position of the tooltip was almost perfect (the overlap between the annotated and the detected bounding box was almost always higher than 50%), detecting the exact position of the tooltip remained a problem for all models (the mAP50:95 score never reached near-perfect levels and average IoU levels were around 80%). Some smoothing of traces of the position of the tooltip may therefore be needed. The issue may in part lie with annotation of the tooltip and the variability of the shape in the image due to rotation of the instrument and the opening and closing of the tooltip. Pose estimation may be a better option in this particular instance (Chen et al., 2020), in which the position of the end of the instrument, the edge of the black shaft and a specific point on the shaft may be used as landmarks. Pose estimation may be better at detecting the centre and the edge of the instrument than object detection.

The confidence provided by the model agreed fairly well with the overlap between the annotated and the predicted bounding box, although it must be said that the overlap between bounding boxes tended to be high overall, and so was confidence. Some more examples of low overlap will be needed to establish a firm association. There were some instances with a high overlap but low confidence and vice versa, so it might be too early to say that confidence can be used to predict overlap when no annotation is available.

The overlap between the annotated and predicted bounding box was only weakly influenced by the position of the instrument in the image. When the image of the object was smaller (i.e. it was further away from the camera), there was more variability in the overlap between the annotated and detected bounding boxes. This observation agrees with previous studies that indicate that YOLO tends to perform worse with smaller objects in the image (e.g. Pham et al. ; He et al., 2020; 2021).

Detection performance did not depend strongly on the pre-trained model (nano, small or medium) or the YOLO version (v3, v5, or v8). Training was fastest for the nano YOLOv8 model, so it is recommended to start with this model. Note that the larger two models (the large and extra-large models) were not tested, and it can therefore not be excluded that performance would have improved for those two models compared to the smaller models. Such testing is expected to benefit from better GPU hardware and is therefore something to consider in future research.

On unseen similar videos the model performed well. The most frequent error was a false negative: No instrument was detected while it was in the display. This error most frequently happened when the tool tip was partially occluded by some of the beads, which is also a known issue for YOLO (e.g. Li et al., 2020; Yu et al., 2022). When there are few failed detections, the position of the target object may be estimated from the position in the frames before and after the frame(s) with the missing values, unless large displacements of the target object can be expected.

The model transfers poorly to the same tool within a different background. This is also a known issue for YOLO (e.g. Li et al., 2022; Pham et al., 2020). This implies that YOLO not only focuses on the target object, but also on how the target object differs in its features from the background. A straightforward way to deal with a change in backgrounds is to train a new model with that new background. If multiple backgrounds are expected, the present results show that a training set should include the expected backgrounds. If annotation of additional images is not possible, data augmentation can be considered in which the original images are transformed to create additional training images using rotation, cropping, or applying filters to the images (e.g. Chung et al., 2020) or by generating additional training images using GANs (e.g. Dewi et al., 2021).

Similar effects of the number of training images and similar performance as for the surgical tool detection was found with low resolution images of a bowl transport task. These results indicate that YOLOv8 handles low resolution images well, and can deal with transparent objects (the bowl) and objects that change shape (hands).

### Conclusion

The present results indicate that excellent object detection can be achieved with a small set of training images. The ease of use of YOLOv8 (Jocher et al., 2023) is likely to mean a paradigm shift in behavioural studies that require video annotation, such as mobile eye tracking studies and studies of object handling. When changing the object or the lab background, it is important to train a new object detector, because performance can be strongly affected. When expecting multiple lab backgrounds, it is important to train a model with examples of all expected backgrounds. The present results do not automatically extend to other computer vision tasks, such as image segmentation and pose estimation. Future studies should examine those tasks separately, but can use the paradigm presented in the present study.

## Footnotes

1 The terms ‘grasper’, ‘tool’ and ‘instrument’ all refer to the same object in the present context. In the surgical community, the term ‘instrument’ is more commonly used.

2 Annotation tends to be faster per object, because the object label does not have to be selected for each bounding box. Moreover focusing on a single object is cognitively less demanding.

3 The actual training time depends on the computational resources available for training, specifically the type and number of GPUs. The same holds for object detection with the trained model.
